# Image Hashtag Recommendations Using a Voting Deep Neural Network and Associative Rules Mining Approach

**DOI:** 10.3390/e22121351

**Published:** 2020-11-30

**Authors:** Tomasz Hachaj, Justyna Miazga

**Affiliations:** Institute of Computer Science, Pedagogical University of Krakow, 2 Podchorazych Ave, 30-084 Krakow, Poland; justyna.miazga@up.krakow.pl

**Keywords:** hashtag recommendations, deep neural network, transfer learning, associative rules mining

## Abstract

Hashtag-based image descriptions are a popular approach for labeling images on social media platforms. In practice, images are often described by more than one hashtag. Due the rapid development of deep neural networks specialized in image embedding and classification, it is now possible to generate those descriptions automatically. In this paper we propose a novel Voting Deep Neural Network with Associative Rules Mining (VDNN-ARM) algorithm that can be used to solve multi-label hashtag recommendation problems. VDNN-ARM is a machine learning approach that utilizes an ensemble of deep neural networks to generate image features, which are then classified to potential hashtag sets. Proposed hashtags are then filtered by a voting schema. The remaining hashtags might be included in a final recommended hashtags dataset by application of associative rules mining, which explores dependencies in certain hashtag groups. Our approach is evaluated on a HARRISON benchmark dataset as a multi-label classification problem. The highest values of our evaluation parameters, including precision, recall, and accuracy, have been obtained for VDNN-ARM with a confidence threshold 0.95. VDNN-ARM outperforms state-of-the-art algorithms, including VGG-Object + VGG-Scene precision by 17.91% as well as ensemble–FFNN (intersection) recall by 32.33% and accuracy by 27.00%. Both the dataset and all source codes we implemented for this research are available for download, and our results can be reproduced.

## 1. Introduction

The number of social media users has continuously increased. Platforms like Facebook, Instagram, Twitter or Flickr are very popular tools for sharing news, keeping in touch with friends, and business promotion. With the aid of Natural Language Processing (NLP), researchers improve methods that might teach Artificial Intelligence (AI) to understand the meaning of messages published in the network. It is still a very challenging task, and algorithms are not perfect in capturing language flexibility, such as sentiments or context of a sentence. Many users include additional information in their post that classifies the context of the message using hashtags. Hashtags are words preceded by the ‘#’ symbol and are used not only to label text data but also images, which is crucial in image-oriented social networks [1]. Hashtags might describe the content of a picture (for example “cat”, “mum”), localization (“downtown”, “beach”), mood (for example “sad”, “happy”), or other topics, even abstract (for example “weather”, “future”, etc.). Users are also able to use different forms of words (“day”, “days”), upper and lowercase letters, slang-inspired words such as “luvu” (which means “love you”), or marketing slogans. The proper choice of hashtags is crucial for correctly categorizing image content and makes an image potentially easier to be found by viewers. In this research we will focus on automatic hashtag generation based solely on image data, formulated as a multi-label classification problem.

### 1.1. State-of-the-Art Works

Hashtags are very popular on social media platforms and impact users’ engagement. As a result, papers devoted to evaluating relationships between message content and hashtags have constantly been proposed by researchers. Authors have investigated relationships between hashtags and posts (text posted by user) [2,3,4], sentiment [5], topic [6], content similarity [7], and so forth.

On many social media platforms the content of the microblogging post is image-oriented. Valuable information can be collected solely from the image. That information might be applicable for recognizing the content of the photo to prepare a set of hashtags that describes the image. As we already mentioned, each image can be described by multiple hashtags (labels). The hashtag should explain and summarize the content and/or meaning of the image. The content might not be identical with the meaning, for example a picture containing a sunset might have hashtags such as “friendship”, “love”, “holidays”, and so forth. As can be seen, it is a very difficult task, even for a human observer, to categorize pictures based only on visual data. What is more, this classification problem is multi-labeled, which means one picture can be assigned to one or more classes.

The common approach for multi-label image classification is deep neural network (DNN)-based hashtag recommendation algorithms. Research on learning and describing the content of images has begun a few years ago [8]; however, hashtag recommendations defined as a multi-labeled problem seems to be a new and not yet explored subject. In a previous paper [1], researchers proposed an open dataset called HARRISON on which one can evaluate the efficiency of the proposed method. The authors also proposed a “baseline” algorithm that utilized a transfer learning approach. An ensemble of deep convolutional neural networks was also used by Reference [9] in a similar task; however, it was a single-label classification problem. A single-label approach was also presented in Reference [10], where the authors explored the possibility of generating hashtags for an input image and leveraged it to generate meaningful anecdotes connected to the essence of the image by applying a character-level Recurrent (RNN) Neural Network [11,12].

Many up-to-date methods utilize additional information besides image data to improve the performance of hashtag recommendation algorithms. The authors of Reference [13] applied transfer learning to train the network to extract embedded images and also used historical tagging information to generate personalized tag recommendations. In Reference [14], overall hashtag recommendations are generated on the basis of both the post’s features from the content modeling module (long short-term memory for text and CNN for images) and the habit influences from the habit modeling module. Another paper [15] proposed methods for calculating the recommended score of each hashtag based on the generated topical representations of multiple features—Distributed Vector Representation of Words, User-Hashtag Matrix, and User-Hashtag Topic Model Based on Short Text Expansion. The authors of Reference [16] proposed a hashtag recommendation algorithm that, besides CNN-based image data embedding, utilized user metadata such as age, gender, home city, and country. The authors of Reference [17] proposed an application of three different pre-trained CNN models to increase differentiation between hashtags. Then, an SVM machine learning model was learned from the extracted features. Semantic embedding modeling for vocabulary (hashtags) expansion is done by the Word2Vec Skip-gram model. The neural image hashtagging (A-NIH) model introduced in Reference [18] consists of two parts—a CNN-based encoder with sequential attention and a gated recurrent units-based decoder for hashtag recommendation. The PhD thesis of Thi Nguyen [19] is a valuable source of up-to-date information in the field of deep learning approaches for recommending image tags.

### 1.2. Motivation of This Paper

As can be seen in the previous section, multi-labeled hashtag recommendations for image data are challenging to model but are a very promising area of research with important applications in the industry, especially on social media platforms. In this research we will propose and evaluate a novel machine learning approach that utilizes an ensemble of deep neural networks to generate images features, which are then classified to a given potential hashtags set. The proposed hashtags are then filtered by a voting schema. The remaining hashtags might be included in a final recommended hashtag dataset by application of associative rules mining, which explores dependencies in certain hashtag groups. This method is called Voting Deep Neural Network with Associative Rules Mining (VDNN-ARM). We evaluated VDNN-ARM on the HARRISON dataset that contains 57383 images in 997 classes (one image can be assigned to more than one class). We implemented and trained other state-of-the-art approaches, namely References [1,9], and our method outperformed those algorithms. Both the dataset and all source codes we implemented for this research are available for download, and our results can be reproduced.

The most important contribution of this paper is the proposition and evaluation of a novel computer method that recommends hashtags from image data. The main novelty of this paper is employing an ensemble of deep neural networks to enhance classification using additional information about dependencies between certain hashtag groups discovered by associative rules mining. To our best knowledge, this combination of deep learning and rules discovering has not been combined into a single voting and recommendation schema for the task of hashtag recommendation. The learning step in our proposed algorithm requires only a training dataset that has a sufficiently large image dataset and information about hashtags associated with them.

## 2. Materials and Methods

In this section we will describe the dataset on which we evaluated our method and schema of our multi-labeled classifier.

### 2.1. Dataset

In this research we utilized a real-world photo dataset, HARRISON [1]. This dataset is composed of 57,383 photos from Instagram. The authors of the dataset processed it by filtering out less frequent hashtags. Finally, each image in the dataset is described with one to ten hashtags. The total number of hashtags is 997, and there is an average of 4.5 associated hashtags for each photo. The task of assigning hashtags to a photo is defined here as multi-label problem because each image might have one or more classes (hashtags) assigned to it. The efficiency of the evaluated method is evaluated using precision calculated on the first suggested hashtag (precision (1)), recall of first five suggested hashtags (recall (5)), and accuracy of first five suggested hashtags (accuracy (5)) as follows:

(1) $$precision\left(k\right)=\frac{results(k)\cap GT}{results(k)}$$

(2) $$recall\left(k\right)=\frac{results(k)\cap GT}{GT}$$

(3) $$accuracy\left(k\right)=\left\{\begin{array}{ccc} 1 & if & result(k)\cap GT\neq \emptyset \\ 0 & if & result(k)\cap GT=\emptyset , \end{array}\right.$$

where *k* is the number of first k “best” (top) hashtags we want to consider, result(k) corresponds to the set of top k hashtags the algorithm predicted, and GT is a set of ground truth hashtags. As can be seen in this evaluation setup, obtaining 100% precision and recall is virtually impossible.

### 2.2. Classifier Architecture

The architecture of our solution was inspired by previous research in this field. Authors have quickly discovered that a single CNN might not be enough to extract all valuable features from an image. In Reference [1], the authors used two deep feature extractors, and both of them had VGG16 architectures [20]; however, the first one was trained on an ImageNet dataset [21] and the second one was trained on a Places dataset [22,23]. Researchers have used very popular transfer learning approaches in which network weights are imported from pre-trained models to extract deep features, and the final classification layers are re-trained using actual classes that are present in the dataset [24,25]. In Reference [1], transfer learning is conducted on the HARRISON dataset. The classification network is composed of two fully connected (dense) layers with ReLu activation and an output layer with a sigmoidal activation function. This is a typical network setup for multi-label classification problems.

The solution proposed in Reference [9] also uses an ensemble of DNNs pretrained on ImageNet, namely VGG16, InceptionV3 [26], and ResNet [27]. Contrary to Reference [1], the transfer learning for each DNN is performed separately on the HARRISON dataset. The solution proposed in Reference [9] is, however, simpler than the one in Reference [1] because the authors considered it a single-label problem, as the output layer for the DNN classification part used softmax. Authors have experimented on various ensemble schemas such as voting, union, intersection, and so forth.

**Figure 1:**
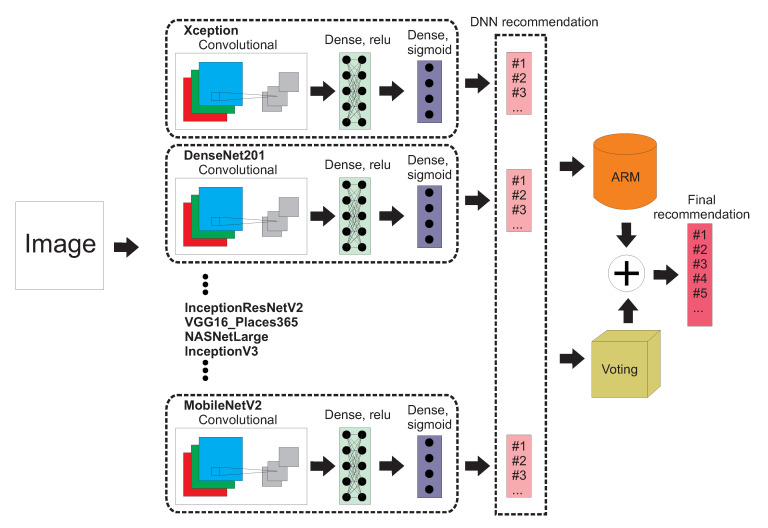
An overview of the Voting Deep Neural Network with Associative Rules Mining (VDNN-ARM) method.

In this paper we propose an approach that incorporates ideas from the above papers, called VDNN-ARM (Voting-based Deep Neural Network architecture with Associative Rules Mining). Figure 1 presents an overview of this method. It consists of several CNN-based feature extractors, namely Xception [28], DenseNet201 [29], InceptionResNetV2 [27], VGG16, NASNetLarge [30], InceptionV3, and MobileNetV2 [31]. Each of these seven networks, besides VGG16, is pretrained on ImageNet; VGG16 is pretrained on Places dataset (the same set as in Reference [1]). Each of those networks accepts input images with dimensions of 224 × 224 in RGB color space. The output of each CNN is processed by a Global Average Pooling 2D layer and then propagated to classification layers. Each of the eight networks has the same classification architecture consisting of two dense layers with 2048 neurons with ReLu activation functions and output layer with sigmoid activation. The role of the last layer is to perform multi-label classification. Similar to Reference [9], we performed separate transfer learning for each of the eight networks on the HARRISON dataset. For a given input image each of the eight DNNs generated sigmoidal output. In Reference [1], the authors classified input images into *x* class labels (they assigned *x* hashtags to an image), which corresponds to *x* top values generated by the output sigmoidal layer. The number of classes/recommendations *x* is arbitrarily decided by the user of an algorithm.

VDNN-ARM takes a different approach in recommending hashtags. Because each DNN generates separate recommendations, we can apply to it various ensemble techniques, similar to Reference [9]. However, besides using only image data during training, we can also utilize information about dependencies between hashtags that are available in the dataset. We can do this, for example, by applying an associative rules mining framework.

Let *T* be a set of all transactions in the given dataset; *A* and *B* are itemsets, and A→B is the association rule [32]. We define the itemset support as counts of *A* among all transactions *T*, in other words the frequency of *A* in a dataset.

(4) $$support\left(A\right)=\frac{\#A}{\#T}.$$

The confidence of association rule A→B is a conditional probability of A given B:

(5) $$confidence\left(A\to B\right)=\frac{support(A\cup B)}{support(A)}.$$

In our case, a transaction is a set of hashtags that describes a given image in our dataset. Because of this, as described in Section 2.1, each transaction in our dataset contains 1 to 10 objects. We want to investigate potential associations between hashtags with reasonable support and confidence. In order to extract frequent itemsets we used the Apriori algorithm [33].

After training the DNN and mining rules from the training dataset, the VDNN-ARM algorithm is applied and described in following section.

#### VDNN-ARM Algorithm

Let us assume there are *l* DNN classifiers. For an input image *I* each CNN $f_{j}$ generates a feature vector that is used as an input to dense (fully connect) the NN with a sigmoid output layer. For the prediction $P_{j,[1\ldots k]}\left(I\right)$ we take *k* classes that correspond to the top values of the NN output layer.

(6) $$P_{j,[1\ldots k]}\left(I\right)=p_{j}{\left(f_{j}\left(I\right)\right)}_{\left[1\ldots k\right]}.$$

In the next step we compose a single vector *P*, which contains predictions from all *l* DNN classifiers.

(7) $$P=[P_{1,[1\ldots k]},\ldots ,P_{l,[1\ldots k]}].$$

Using *P* we generate two vectors: *C*, which contains unique elements from *P*, and $C_{fr}$, which contains counts of hashtag class labels from *C* in *P*.

(8) $$\left\{\begin{array}{c} C=[h_{1},h_{2},\ldots ,h_{n}]\\ C_{fr}=\left[\#h_{1},\#h_{2},\ldots ,\#h_{n}\right], \end{array}\right.$$

where $\#h_{1}\geq \#h_{2}\geq \ldots \geq \#h_{n}$, $h_{i}$ is a hashtag class label, and $\#h_{i}$ is counts of the hashtag class label $h_{i}$ in *P*.

Then, we perform thresholding of *C* and create two vectors: $C_{1}$, which contains classes that appeared in more than one classifier output, and $C_{2}$, which contains those appearing in only one output. $C_{1}$ is then ordered by descending number of hashtag counts.

(9) $$\left\{\begin{array}{c} C_{1}=\left[h_{1},h_{2},\ldots ,h_{m}\right]\\ C_{2}=\left[h_{m+1},\ldots ,h_{n}\right]\\ , \end{array}\right.$$

where $\#h_{m}\geq 2$

$C_{1}$ contains classes for which at least two classifiers have voted. Then, we apply associative rules mining for $C_{1}$ and generate $ARM(C_{1})$, which is a set of all conclusions supported by associative rules. Then, we take the common part of $ARM(C_{1})$ with C2. In hashtag set $C_{3}$ we have only classes that appeared as a result of rules supported by $C_{1}$ and which are also present in $C_{2}$.

(10) $$C_{3}=ARM\left(C_{1}\right)\cap C_{2},$$

where ARM is reasoning applied by associative rules mining (ARM) on the rules we have previously discovered.

Classes that were present in $C_{3}$ are assigned at the end of vector $C_{1}$. *S* is a vector that contains the suggested hashtags for image *I* ordered from most frequently proposed by classifiers to those that appeared only once, but they were supported by ARM.

(11) $$S=[C_{1},C_{3}].$$

Now we can take *x* first elements from *S* to generate *x* top hashtag suggestions for a given image.

## 3. Results

We implemented our approach in Python 3.6. Among the most important packages we used were Keras 2.4.3 and Tensorflow 2.3.1 for DNN implementation and GPU-accelerated tensor calculations. We used pre-trained CNN network weights from Keras-Applications 1.0.8 that were trained on the ImageNet dataset [21]. We also used VGG16 network weights [23] that were trained on the Places dataset [22]. For associative rules mining we utilized the mlxtend 0.17.3. package [34]. To evaluate the proposed method we used the HARRISION dataset [1] described in Section 2.1. We used 52383 randomly chosen objects in the training set and 5000 in the validation dataset.

All classifiers have been trained using a first-order gradient-based Adam optimizer [35] with a binary cross-entropy loss function.

In order to generate associative rules we set the minimal support threshold in the a priori algorithm to 0.0001. We filtered out all rules with confidence below 0.001.

We also implemented and trained algorithms proposed in References [1,9]. In the case of Reference [9] we replaced the softmax layer with a dense layer with sigmoidal activation function to make this classifier applicable to multi-label problems. All source codes we implemented in our research can be downloaded from github (https://github.com/browarsoftware/VDNN-ARM). Calculations were performed on a PC computer with Intel i7-9700 3.00 GHz CPU, 64 GB RAM running Windows 10 OS. We used NVIDIA GeForce RTX 2060 GPU.

**Figure 2:**
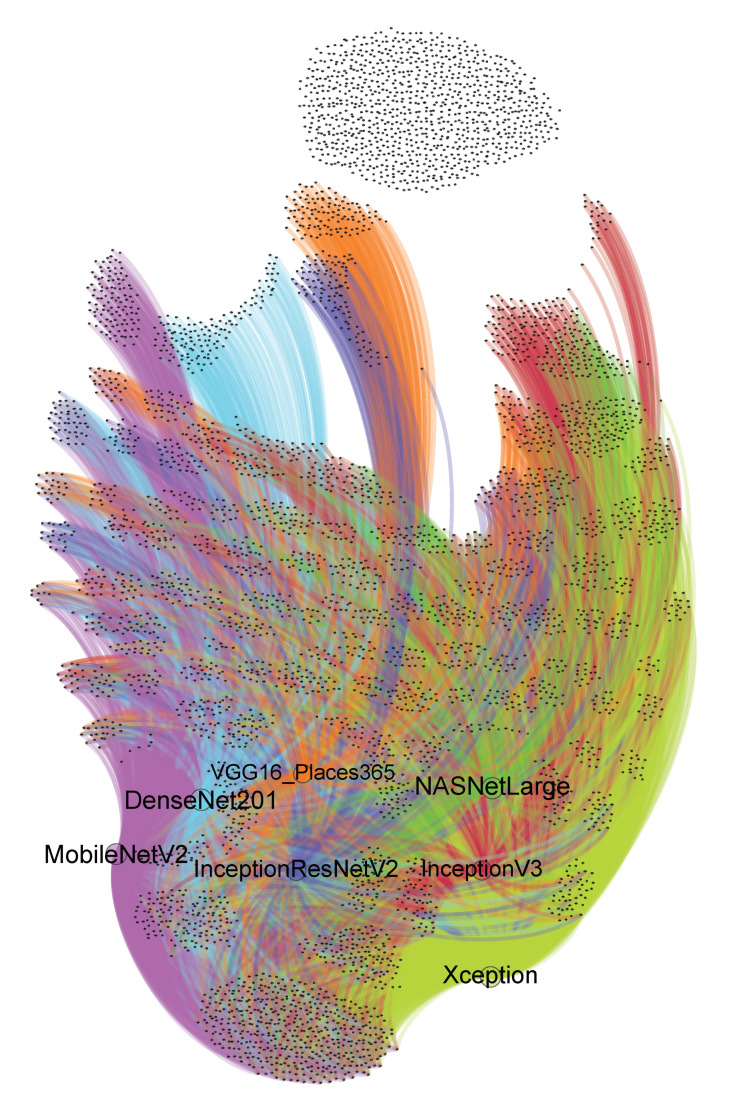
Graphic representation of accuracy (5) tests on each DNN network.

In Figure 2 we present the accuracy (5) tests on each DNN network in the form of a graph. We generated this visualization using Gephi 0.9.2 software [36]. Graph layout was generated using the ForceAtlas2 algorithm [37].

**Figure 3:**
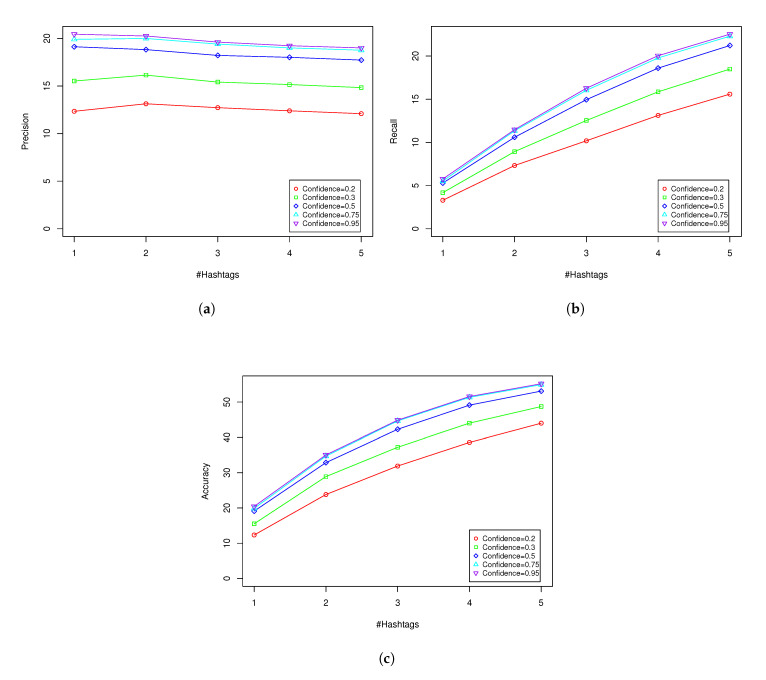
Graphical results of Table 1. Plot (**a**) shows precision obtained for various numbers of hashtags and confidence of ARM. Plot (**b**) visualizes recall and (**c**) accuracy.

Each node (vertex) represents an image from the validation dataset. If an image is connected to a node by a colored edge, this represents a particular DNN network, which means that at least one of the top five hashtags generated by that DNN is among hashtags describing this image. An image might have several connections to different vertices if, and only if, it has correctly passed the accuracy (5) test in more than one network. If the node is isolated, that means it has not been correctly classified by any network. As can be seen, there is a group of images that are not correctly classified by any network. They are visible in the top part of the graph as isolated grey points. This clearly shows there are some limitations to algorithms that are based on applying DNN to hashtag discovery that cannot be overcome. In addition, various DNN covers are not identical subsets of all images. This means applying an ensemble of several DNNs of various types might give better results than using only a single DNN. In Table 1 and in Figure 3 we present detailed results of the proposed algorithm with various threshold values of confidence for ARM. It is also possible that our proposed method will not generate hashtag recommendations. This might happen when C1=∅ (see Equation (9)). Column #Recommended hashtags means that we evaluate precision, recall, and accuracy for no more than *x* top generated hashtags, where *x* is not more than length of vector *S* (see (11)). “No restrictions” means that we calculate all evaluation parameters for the whole vector *S*. The bold font indicates parameters with the highest values in the table. The best results were obtained for VDNN-ARM with threshold = 0.95 if we take into account a limited number of hashtags. When there is no restriction on the number of hashtags, the highest recall and accuracy were obtained for VDNN-ARM with threshold = 0.2. In Equation (7) we use parameter k=5, the same as in References [1,9].

In all cases, when the number of proposed hashtags increases, the precision decreases, and the recall and accuracy become higher. This is an expected behavior. At the beginning of vector *S* there are the most voted (probable) hashtags. When the number of considered hashtags increases, the denominator in the precision equations also increases, and more and more less probable hashtags are included in the evaluation. In the case of recall and accuracy, the higher number of hashtags causes an increase in the numerator, which increases recall and accuracy. When we do not limit number of hashtags to 5 (“No restriction”), the recall and accuracy achieve the highest value.

Table 2 presents a comparison of the proposed method to state-of-the-art approaches. The highest value for all coefficients were obtained for VDNN-ARM with a confidence threshold 0.95. VDNN-ARM outperforms the precision (1) of VGG-Object + VGG-Scene [9] by 17.91%; in the case of Ensemble–FFNN (intersection) [9], the recall (5) increased by 32.33% and accuracy (5) by 27.00%.

## 4. Discussion

As can be seen in Section 4, the proposed method outperformed state-of-the-art approaches. Due to its voting schema this method incorporates benefits of both union and intersection schema. An intersection schema is responsible for aggregating and counting the recommended hashtag label results of each sub-DNN network. Union does not exclude less frequent hashtags from the final recommendation. Application of associative rules mining utilizes additional knowledge about conditional dependencies between hashtags. As can be seen in Table 2, in the case of algorithm [1], the image content data solely generated by DNN is not enough to overcome baseline results. The transfer learning approach utilized features of CNN for successful classification of image content.

Typically, in up-to-date literature, authors use methods that suggest *x* most probable hashtags from an output sigmoidal layer, where *x* is arbitrarily chosen by the user. VDNN-ARM allows one to manually choose the number of hashtag recommendations; however, by applying ARM and confidence thresholding schema, it might also be used to include less frequent hashtags that are recommended by ARM. The highest values of evaluation parameters, including precision (1), recall (5), and accuracy (5), have been obtained for VDNN-ARM with confidence threshold =0.95. All parameters decreased as the confidence threshold decreased (see Table 1). This result suggests that increasing the confidence of ARM rules results in an increase in classifier performance. This very important indicator suggests that applying a higher number of confident rules leads to a higher number of “matching” hashtags generated by the approach.

Our results suggest the proposed algorithm is a promising approach that can be successfully applied in practice. Another important find is the limitations of DNN-based hashtag discovery algorithms, which we discussed in Section and visualized in Figure 2. In order to improve the evaluation results we need to improve other parts of algorithm than CNN-based image feature extractors. According to Reference [38] there is a certain category of image hashtags that authors named “stophashtags”. This name is inspired from the term “stopwords”, which is used in the field of computational linguistics to refer to common and non-descriptive words found in almost every text document. Authors of that research have shown that, contrary to descriptive hashtags (hashtags relevant to the subject of an image), “stophashtags” are characterized by a high normalized subject (hashtag) frequency on irrelevant subject categories. Because we used a third-party benchmark dataset in this research, which has already been preprocessed by their creators and used in other research, we did not filter out potential “stophashtags”. It is possible that filtering “stophashtags” might improve the results of our method; however, the algorithm described in Reference [38] should operate on all acquired hashtags, not a subset that is present in the HARRISON dataset. In our future research in the field of hashtag recommendations, we plan to acquire an even larger dataset than HARRISON and apply to it “stophashtags” filtering. We believe this operation might lead to even more interesting and valuable results.

## 5. Conclusions

The proposed VDNN-ARM hashtag recommendation algorithm is an efficient approach that can be applied to any type of social media image data. As can be seen, the precision (1) coefficient is still relatively low; the first top hashtag appears only in about one-fifth of validation data. In the case of accuracy (5), over 55% of validation data has at least one correctly assigned hashtag; this is because the multi-label classification problem is difficult to correct: not only are there 997 classes, but the ground truth of class labels might not match the objects, scenes, and places that are present in images. Real-life hashtag descriptions often tell the state of mind, sentiment, or some abstract context that the person had in the moment of taking or publishing a photo. Each photo might have between 1 to 10 different hashtags, and that number varies between images. Therefore, when we do not have additional knowledge about the context of the picture (so called “a story behind photo”), we might not be able to mine/learn the rules that govern certain phenomena. The knowledge about those rules is also not fully understandable to a person who might try to manually assign hashtags. Because of this, it is very improbable that any algorithm based on HARRISON data will obtain perfect or even nearly perfect accuracy. Contrary to already published methods, our algorithm is capable of limiting the number of proposed hashtags by applying the ARM approach and its thresholding schema. Thanks to this, the VDNN-ARM in no-restriction mode can easily trade-off between precision and accuracy/recall.

We believe our algorithm is not limited to hashtag recommendation; it can be applied to any type of multi-labeled image classification data. In our opinion, the next step in research should be developing methods that utilize additional information, such as the context of the photo, which can be extracted from discussions about this photo on social media, geopositioning information, and so forth. These additional data, besides image data and baseline hashtag information, seem to be crucial to increase the efficiency of multi-label hashtag recommendations above a certain level limited by image-oriented DNNs. The limitations of image-oriented DNNs are clearly visible in Figure 2.

## Figures and Tables

**Table 1:** Results of precision, recall, and accuracy obtained by VDNN-ARM depending on the confidence value.

ARM Confidence	#Recommended Hashtags	Precision	Recall	Accuracy
≥0.2	No more than 1	12.34	3.30	12.34
	No more than 2	13.13	7.32	23.82
	No more than 3	12.72	10.18	31.90
	No more than 4	12.39	13.12	38.58
	No more than 5	12.09	15.58	44.00
	No restrictions	11.59	**33.67**	**67.16**
≥0.3	No more than 1	15.52	4.18	15.52
	No more than 2	16.14	8.93	28.90
	No more than 3	15.41	12.53	37.22
	No more than 4	15.15	15.85	44.02
	No more than 5	14.83	18.48	48.72
	No restrictions	14.49	30.12	63.24
≥0.5	No more than 1	19.12	5.29	19.12
	No more than 2	18.82	10.59	32.86
	No more than 3	18.21	14.94	42.28
	No more than 4	18.01	18.59	49.12
	No more than 5	17.72	21.21	53.10
	No restrictions	17.43	26.77	59.44
≥0.75	No more than 1	19.90	5.52	19.90
	No more than 2	19.99	11.34	34.70
	No more than 3	19.40	16.02	44.64
	No more than 4	19.00	19.77	51.32
	No more than 5	18.76	22.29	54.92
	No restrictions	18.48	25.73	58.72
≥0.95	No more than 1	**20.44**	5.76	20.44
	No more than 2	20.25	11.47	35.04
	No more than 3	19.61	16.26	44.86
	No more than 4	19.23	20.03	51.56
	No more than 5	19.00	**22.52**	**55.18**
	No restrictions	18.77	25.45	58.46

**Table 2:** Comparison of precision (1), recall (5), and accuracy (5) of state-of-the-art algorithms and VDNN-ARM.

Method	Precision (1)	Recall (5)	Accuracy (5)
VGG-Object + VGG-Scene (Baseline) [1]	16.78	13.33	35.80
Ensemble − FFNN (union) [9]	11.36	13.48	37.46
Ensemble − FFNN (intersection) [9]	14.12	15.24	40.28
VDNN-ARM confidence = 0.2	12.34	15.58	44.00
VDNN-ARM confidence = 0.3	15.52	18.48	48.72
VDNN-ARM confidence = 0.5	19.12	21.21	53.10
VDNN-ARM confidence = 0.75	19.90	22.29	54.92
**VDNN-ARM confidence = 0.95**	**20.44**	**22.52**	**55.18**
